# Complex neural codes in rat prelimbic cortex are stable across days on a spatial decision task

**DOI:** 10.3389/fnbeh.2014.00120

**Published:** 2014-04-23

**Authors:** Nathaniel J. Powell, A. David Redish

**Affiliations:** ^1^Graduate Program in Neuroscience, University of MinnesotaMinneapolis, MN, USA; ^2^Department of Neuroscience, University of MinnesotaMinneapolis, MN, USA

**Keywords:** prelimbic cortex, decision-making, rats, neural ensemble data, tetrode recording, prefrontal cortex (PFC), behavior, animal

## Abstract

The rodent prelimbic cortex has been shown to play an important role in cognitive processing, and has been implicated in encoding many different parameters relevant to solving decision-making tasks. However, it is not known how the prelimbic cortex represents all these disparate variables, and if they are simultaneously represented when the task requires it. In order to investigate this question, we trained rats to run the Multiple-T Left Right Alternate (MT-LRA) task and recorded multi-unit ensembles from their prelimbic regions. Significant populations of cells in the prelimbic cortex represented the strategy controlling reward receipt on a given lap, whether the animal chose to go right or left on a given lap, and whether the animal made a correct decision or an error on a given lap. These populations overlapped in the cells recorded, with several cells demonstrating differential firing to all three variables. The spatial and strategic firing patterns of individual prelimbic cells were highly conserved across several days of running this task, indicating that each cell encoded the same information across days.

## Introduction

The rodent prelimbic cortex (PL) plays an important role in cognitive processing and the solving of decision-making tasks (Kolb, 1990; Dalley et al., 2004; Kesner and Churchwell, 2011) including working memory (Yoon et al., 2008; Horst and Laubach, 2009), interval timing (Kim et al., 2009; Narayanan and Laubach, 2009), the encoding of uncertainty (Karlsson et al., 2012), reward receipt (Pratt and Mizumori, 2001), and behavioral strategy (Jung et al., 1998; Peyrache et al., 2009; Rich and Shapiro, 2009; Benchenane et al., 2010; Durstewitz et al., 2010). Other studies have found prelimbic neural correlates encoding information about recently encountered errors while performing a decision task (Narayanan et al., 2006; Narayanan and Laubach, 2008) and shown that neurons in PL have non–uniform firing patterns over space (Jung et al., 1998; Hok et al., 2005; de Saint Blanquat et al., 2010).

How PL satisfies all of these different roles remains unclear. Do they co-occur on a single task? Are the different firing correlates particular to the task, or could several of them be observed on the same task if required? And do these different firing correlates arise from different populations of cells, or do the populations somehow overlap? We tested these questions by recording from the PL of rodents as they attempted to solve a spatial decision-making task.

**Figure 1:**
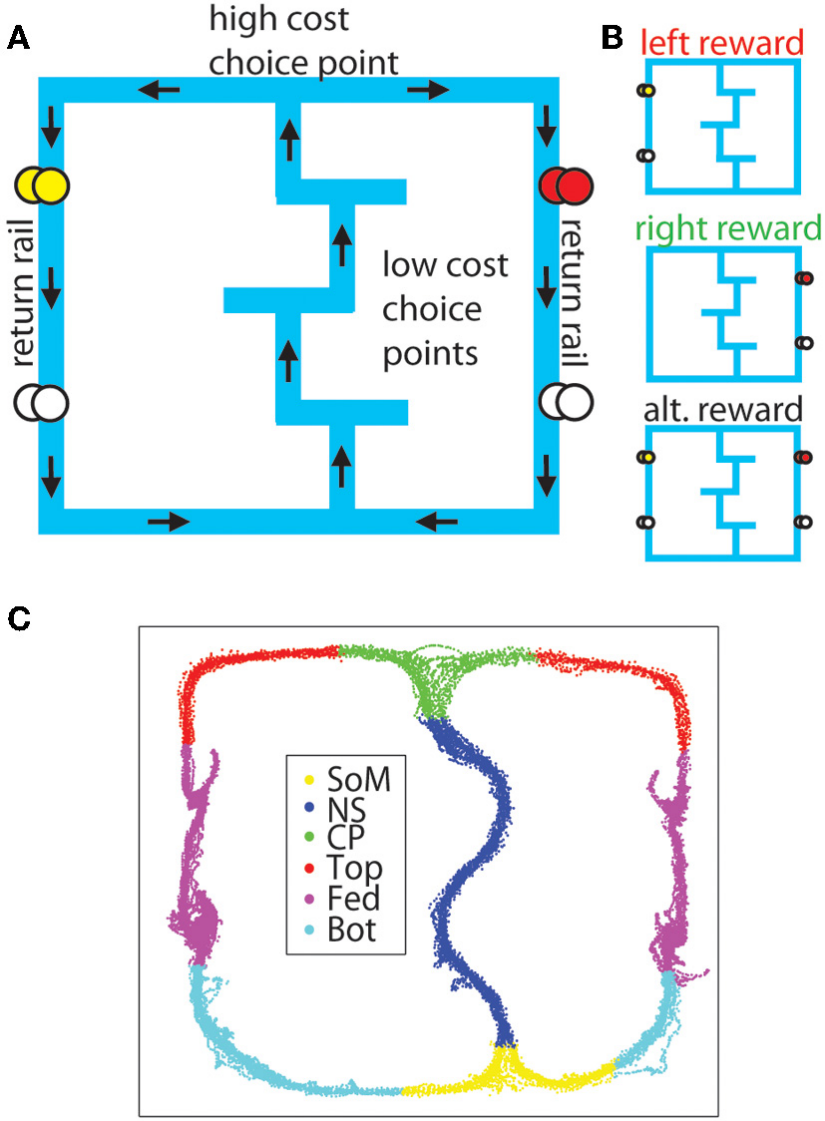
**The MT-LRA task. (A)** The task consists of a central track containing three low-cost T-choices, a high-cost T-choice at the top of the central track, and two return-rails on which reward was delivered under either *leftward*, *rightward*, or *alternating* contingencies. The first reward site on the left side provided banana-flavored food pellets, while the first reward site on the right side provided fruit-flavored food pellets. The second reward site on both sides provided unflavored (white) food pellets. **(B)** Reward was only delivered if the animal made the correct choice under the active contingency. **(C)** For analysis, the maze was divided into six sections (SoM, start of maze; NS, navigation sequence ; CP, choice point; Top, top rails; Fed, feeder sites; Bot, bottom rails).

The Multiple-T Left Right Alternate task (MT-LRA) is a multiple-choice, sequential decision task which allows us to spatially differentiate low cost choices, high cost choices, and the reward location (Gupta et al., 2010; Blumenthal et al., 2011; Steiner and Redish, 2012, see Figure 1). This task has a number of features which will allow us to study these different components: It is a spatial task, in which different strategic components need to be active on different parts of the maze, allowing us to identify intra-lap strategies (akin to that of Junget al., 1998), and compare them to spatial firing patterns (akin to that of Hok et al., 2005; de Saint Blanquat et al., 2010). It contains three strategies occurring on the same maze and a 6 day “switch sequence” which involves unannounced switches in the reward contingencies that the animals must follow in order to receive reward, allowing us to identify strategy- and strategy-switch-related encoding (akin to that of Peyrache et al., 2009; Rich and Shapiro, 2009; Benchenane et al., 2010; Durstewitz et al., 2010). Finally, it forces animals to run through the central track on every lap which should produce similar behavior, allowing us to control for subtle differences in posture that have been previously identified as potential confounds in identifying prefrontal neural correlates (Euston and McNaughton, 2006; Cowen and McNaughton, 2007).

We recorded neural ensembles (up to 30 neurons simultaneously) from 3 rats through the 6-day strategy-switch sequence. Using established techniques (Schmitzer-Torbert and Redish, 2004; Tolias et al., 2007), we were able to identify 60 single neurons recorded across at least 2 days, including 8 neurons recorded across all 6 days. Individual neurons in rat prefrontal cortex encoded all of the different aspects noted above on a single task; these multiple-aspect-encodings were consistent across days, implying a global, but consistent, representation of task information in rat prefrontal cortex.

## Methods

### Animals

3 male Fisher Brown Norway (FBNF-1) rats aged 8-12 months at the start of behavior were used in this study. Animals were housed on a 12 h light-dark cycle and all experiments for a given rat were run at the same time each day during the rat's light phase. Prior to task training, animals were handled and trained to eat the food pellets used as reward on the task. During training and recording periods, animals received all of their food during behavior or during handling immediately afterward. Animals had free access to water in their cages throughout the experiment. The rats' weights were monitored daily and maintained at or above their 80% free food weight by hand-feeding after running if necessary. All training procedures were approved by the Institutional Animal Care and Use Committee at the University of Minnesota and in accordance with the National Institutes of Health guidelines.

## Data acquisition

### Behavior

The Multiple-T-LRA task was run on a raised linear track (see Figure 1A). The track consisted of a Sideways-8 topology, in which rats ran along a central track before arriving at a high-cost choice point at which they had to turn left or right. If they made the correct decision for the active reward contingency, they received rewards at two feeder sites along the return rail. If they made the incorrect decision, they received no rewards and had to continue past the inactive feeders to make another circuit through the central track. These low-cost T-choices along the central track were changed pseudo-randomly for each session to produce a sequence of turns the animal had to navigate in order to reach the fourth (high-cost) choice. We refer to the sequence of three internal (low-cost) T-choices as the *navigation sequence* (NS). The fourth (high-cost) T-choice is referred to as the *choice point* (CP).

A daily session on the task began when the rat was placed at the base of the first T on the bottom rail of the track (*Start of Maze* or SoM). The animal proceeded up the navigation sequence until he reached the high-cost choice point at the top rail. After making his decision, the rat proceeded across the top rail toward one of the return rails. The return rails each had two feeder locations along their length; The first feeder (Med-Associates, St Albans, VT, USA) delivered two flavored food pellets (45 mg each, Research Diets, New Brunswick, NJ, USA, fruit flavor on the right and banana flavor on the left) and the second feeder on either side delivered two unflavored (white) food pellets. After passing the feeder locations, the animal ran along the bottom rail of the track back to the start location (SoM) from which he could begin another lap. Animals ran laps continuously for one 40 min session each day throughout the training and experimental parts of this task with no upper limit on the number of laps or pellets per session. On average, animals ran 43 laps and received 142 pellets (6.4 g) per session for the switch sequence (described below). Animals were post fed pellets after sessions as necessary to ensure they did not drop below 80% of their free food weight.

Pellet rewards at the feeder locations were provided if the animal went to the correct side as defined by the current reward *contingency*. There were 3 possible reward contingencies, Left (L) where only the left feeders were rewarded, Right (R) where only the right feeders were rewarded, and Alternating (A) where the feeders on the side opposite to the previous lap were rewarded. (The first lap under the alternating (A) condition was always rewarded, but subsequent laps were only rewarded if the animal went to the opposite side as the previous lap, i.e., alternating left-right-left, etc.) Throughout training, one reward contingency was active per session and the active contingency varied psuedo-randomly between days. Once animals were ready for data collection, we began a *switch sequence*, in which the reward contingency was changed halfway through the session. (The actual contingency-switch time occurred randomly between 18 and 22 min into the 40 min session.) No external cue was provided to predict the switch. The switch sequence lasted 6 days so that every possible combination of initial and final contingency could be presented to the animal, i.e., L to R, L to A, R to L, R to A, A to L, and A to R. The order in which these contingencies were presented was randomized between animals.

The Multiple-T and Multiple-T-LRA tasks have been used by many previous studies (Schmitzer-Torbert and Redish, 2004; Gupta et al., 2010; van der Meer et al., 2010; Blumenthal et al., 2011; Steiner and Redish, 2012) and some aspects of its design (including the use of multiple feeders on the return rails and different flavors at different feeder locations) were maintained in this experiment for the sake of consistency with these previous studies, even though these specific aspects of the design were not important for the analyses conducted in this experiment.

### Behavioral training

Initial training of the animals was conducted with one of the sides blocked off at the start of maze and choice point, so the animals were forced to run laps around a single loop to either the left or right. Animals ran 40 min sessions to both left and right on blocked tracks for 1–2 weeks until they had run over 50 laps in a session on both the left and the right sides. For these sessions, the available feeders were always rewarded when the animal completed a lap. Once they had reached this 50 lap criterion, the blocks were removed and the animals continued training on the open track initially with just the L and R contingencies pseudo-randomly chosen for each day. Once the animals were running over 50 laps per day and over 80% correct laps, they were introduced to the A contingency as well. When animals were running all 3 contingencies consistently with more than 80% of laps correct and 50 or more laps per session, they underwent the surgical implantation of a 12-tetrode micro-array drive targeted to the pre-limbic region of cortex (PL). Following surgery, the animals were allowed to recover for 2–4 days before returning to running on the track. When performance had returned to previous levels, the tetrodes had been advanced to the recording locations, and the cellular ensemble had been maximized, we began the switch sequence. This paper contains only data recorded during the 6 day switch sequence for these 3 rats.

### Surgery

After training, animals were implanted with 12-tetrode hyperdrives (Kopf). Anesthesia was initiated with sodium pentobarbital (Nembutal, 50 mg/kg, delivered IP) and maintained while on the stereotax via isoflurane mixed at 0.5–2% into medical grade oxygen delivered via a nosecone. Two different implantation techniques were utilized for the hyperdrive itself. The first rat (R193) was implanted with a single bundle drive targeted to the right pre-limbic and infra-limbic (IL) regions of mPFC using a technique based on the one described by Euston and McNaughton (2006). A craniotomy was opened at AP + 3.0 mm from Bregma, ML 1.3 mm, and the drive bundle was angled 9.5° toward the midline, allowing the bundle to avoid the sagittal sinus and the corpus callosum.

**Figure 2:**
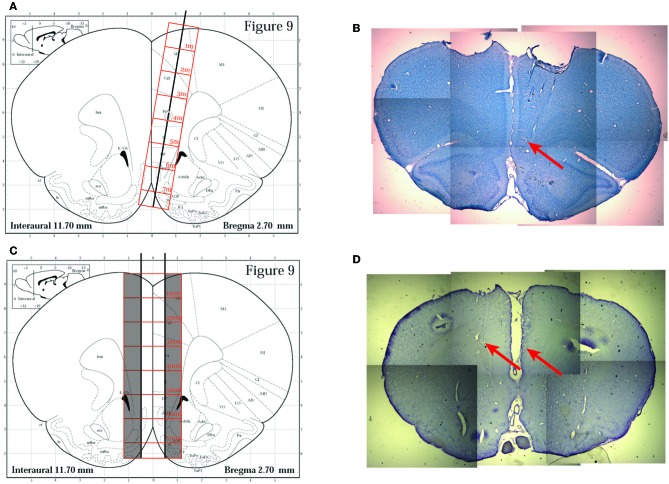
**Histology**. **(A)** The original implantation technique used for the first rat (R193) based on the approach described in Euston and McNaughton (2006). A single bundle drive was implanted laterally and angled toward the midline. Image modified from Paxinos and Watson (1996). **(B)** A representative histology slice from rat R193 showing tetrode tracks and gliosis scars (red arrow). **(C)** The implantation technique utilized for the other two rats (R195 and R199). A dual bundle drive was implanted on either side of the saggital sinus. Image modified from Paxinos and Watson (1996). **(D)** A representative histology slice from R195 showing gliosis scars (red arrows) indicating final recording cites.

For the subsequent two rats (R195, R199), we utilized a surgical technique we developed specifically for these experiments. We implanted a dual bundle drive (1 mm spacing between bundles) with 6 tetrodes and 1 reference electrode targeted to each hemisphere of the PFC. The bundle was designed to be implanted on both sides of the saggittal sinus, which was localized by opening a large craniotomy at AP + 3.0 mm from Bregma and extending approximately 1.4 mm laterally on either side of the midline and approximately 1.4 mm A-P. We created this craniotomy by slowly grinding away the skull with a burr in a high speed drill (Foredom, 45,000 RPM) until we could visualize the central sinus, then we were able to carefully remove the final layers of skull and dura matter over the brain on either side and target each of our bundles to one side of cortex. Tetrodes were advanced at least 2 full turns (640 μm) as soon as possible following surgery (~15 min after removal from the stereotax), and were advanced every day subsequently until the switch sequence began. Recording locations were verified histologically to be in the inferior prelimbic cortex, see Figure 2.

In each of these surgeries, a ground screw was secured to the skull along with at least 6 anchor screws, and the drive was held in place by a dental acrylic base secured to the anchor screws. All animals were given a 3-day course of antibiotics (Baytril) and returned to free food following surgery for several days until they were ready to return to running on the track.

### Data acquisition

Prior to surgical implantation, the animal's position on the maze was tracked via an overhead camera from an LED light on an in-house-designed backpack. After surgery, the rat's position was tracked by LEDs mounted on the headstage plugged into his implant. Local field potentials and unit spiking activity were recorded on a 64-channel analog Cheetah recording system (Neuralynx, Bozeman, MT, USA). Spikes were identified and recorded online using built in filters, then were sorted into individual units offline. Pre-clusters were formed automatically using KlustaKwik (KD Harris), then sorted into individual putative units with the MClust 3.5 software package (AD Redish). Cellular identity and spiking times were registered to the animal's position on the maze and feeder food delivery events that were recorded by the Neuralynx software. A total of 330 cells were identified, predominantly from the inferior PL. Based on assessment of tetrode depths, ~10% of cells may have come from superficial IL, but no clear distinctions were seen. Additionally, based on firing rate measures, few putative interneurons were recorded (less than 10%). Accordingly, all neurons were pooled for all analyses. Subsequent analysis (see below) revealed that many of these cells were instances of the same cell being recorded across multiple days. Waveform matching analyses (see below) left us with 205 putative individual cells.

## Data analysis

### Lap analyses

For analysis purposes, the track on which the animal ran was divided into 6 sections (SoM, NS, CP, Top, Fed, Bot) defined by pixel coordinates relative to experimenter identified locations in the animal's tracking data and the zones used to automatically trigger the feeders in the experiment (see Figure 1).

A lap was defined as a complete cycle from SoM back around to SoM. Animals always started the session at the SoM for their first lap, but the end of the session occurred whenever the 40 min time expired, so animals may not have made it all the way back to SoM on their last putative lap. If the animal made it from SoM to at least the first feeder on their last journey of the session, it was considered to be the last lap. Conversely if they did not make it as far as the first feeder the journey was not counted as a lap. Laps were labeled as *correct* or *error* and *left* or *right* based on the animal's choices. The switch time (which was randomized for each session within a 4 min window from 18–22 min into the session) was recorded for each session, and laps were labeled as being *before* or *after* the switch based on which strategy dictated reward receipt on that lap (the pre-switch strategy or the post-switch strategy, independent of the animal's choice).

Each cell's firing rate was determined for each maze section on each lap by counting the number of spikes recorded during that section divided by the time elapsed during that section, and the *z*-scored firing rate for a particular lap was determined by taking the difference of the firing rate of that cell in that section on that lap from the mean firing rate of that cell in that section for that day, divided by the standard deviation of the firing rate of that cell in that section on that day.

### Measuring changes relative to task parameters

We measured differential responses to binary task parameters (going left vs. going right, strategy before vs. strategy after the switch, choosing correctly vs. choosing incorrectly) in two ways.

### *z*-score analyses

We measured the firing rate difference between the average firing rate for the two components and divided it by the standard deviation of one of the two components. e.g.,

(1) $$z_{B}(A)=\frac{F_{A}-\mu (F_{B})}{\sigma (F_{B})}$$

Where *F*_*A*_ is the firing rate on lap type A, μ(*F*_*B*_) is the mean firing rate from laps of type B, and σ(*F*_*B*_) is the standard deviation of the firing rate from laps of type B. Because the standard deviations were not equal, *z*_*B*_(*A*) was not necessarily equal to *z*_*A*_(*B*), and these analyses were performed in each direction separately.

This pseudo-*z*-score was calculated for each of the three effect pairs (correct/error, left/right, before/after). To compare the distribution of observed pseudo-*z*-scores to the expected random distribution, the psuedo-*z*-scores were calculated as above, but the labels of the laps (before/after, left/right, correct/error) were shuffled randomly. This shuffling process was repeated 1000 times, then the resulting distribution was fit to a gaussian model, and plotted for comparison to the actual recorded distribution of pseudo-*z*-score values.

### KS-tests

As an alternate analysis capable of directly comparing the firing of a cell in response to the effect pairs described above, we used a Kolmogorov-Smirnov test to determine whether the distribution of average firing rates on laps identified in one category was different from that of the other. We counted the number of cells with significant (α = 0.05) distributions to the binary pair. We compared this count to the expected count from two controls, an ISI-shuffled control (ISI) and a strategy-shuffled control (ID). To generate the ISI-shuffle, we randomly reordered the inter-spike intervals of each individual cell and recalculated the KS-test significance, counting the number of cells with a significant difference between the binary alternatives using firing rates generated from shuffled ISIs. To generate the strategy-shuffled control, we took the original firing rates, but changed the identification of which lap belonged to which category randomly. Again, we recalculated the cells with a significant difference as measured through a KS-test. In both cases we calculated the mean and standard deviation of the expected number of significant cells from a distribution of 1000 different random shuffles. We used a *Z*-test to determine whether the number of cells showing differential firing in the actual population of cells was significantly larger than the distribution of expected numbers recorded from each random shuffle case.

### Path differences control

Previous research (Euston and McNaughton, 2006; Cowen and McNaughton, 2007) has shown that some differential firing patterns observed in rodent mPFC can be accounted for by subtle differences in the movements of the animal. On our spatial task, positional differences are to be expected between most sets of laps because laps will be run to different sides of the track. However, on the navigation sequence of our track the animal's path from one lap to the next should be similar. We therefore attempted to assess whether the cells we discovered on this region of the track that encoded strategy laps before vs. after the switch, left-going vs. right-going laps, or correct vs. error laps could be explained by spatial differences. We binned the spatial position of the animal on all laps through the navigation sequence into a 2-dimensional grid. For each session on the track, we then calculated the average occupancy of this grid for laps belonging to one side of the effect pairs described above to the occupancy on laps belonging to the other side of the effect pair, then we calculated the correlation coefficient for these two average occupancies (i.e., we correlated the occupancy of all laps before the switch to the occupancy of all laps after the switch). This correlation coefficient should be inversely related to any path differences. We compared the correlation coefficients to a distribution of correlation coefficients calculated from shuffling the identity of individual laps and correlating the two groups, and repeated our analyses after removing all sessions where there was a significant difference in behavior on the NS portion of the maze (before vs. after switch *n* = 6 sessions, left vs. right *n* = 5 sessions, correct vs. error *n* = 1 session). Removing these sessions made no qualitative difference in our results.

Additionally, we controlled for running speed differences by fitting a robust regression of normalized running speed in each section of the maze to the recorded firing rates in that section of the maze over all laps. Any cells that were found to have a significantly non–zero regression coefficient were removed from the count of cells with significant differential firing as assessed by the KS-test method described above.

A similar control was conducted to remove the effect of cells with a continuously variable firing rate over time from the before vs. after switch selective cells. A robust regression of firing rate vs. lap number was fit to all cells, and cells with a significantly non–zero regression coefficient were removed from the count of cells with significant differential firing as assessed by the KS-test method described above.

### Position decoding

In order to analyze spatial information through decoding, positions were first linearized separately for the left and right loops from start of maze back to start of maze. Spatial extent was normalized between key landmarks (the start of maze, the choice point, feeder1, feeder 2, and the start of maze again at the end of the lap). Paths were projected onto this linear coordinate system as per previous studies (Schmitzer-Torbert and Redish, 2004; Gupta et al., 2010).

Bayesian decoding was conducted according to standard methods (Zhang et al., 1998). For each session, we divided the total set of laps randomly into evenly sized training and test sets, binned the spatial locations into 25 spatial bins, used the tuning curves calculated from the training set to train the decoder, and calculated the likelihood of decoding to each spatial bin for each 500 ms temporal bin of the test set. We repeated this process 100 times (with different randomly chosen training and test sets each time). These multiple random samples were then averaged first within rat and then between rats to get the final decoded probability for both left and right laps. The linearized decoding for both left and right laps were visually nearly identical, so these paths were then averaged to produce the plots seen in Figure 10.

The same analyses were conducted on example hippocampal cells recorded from a different group of rats run on the same task. The data collection, processing, and behavior of these rats were equivalent to the rats presented here, but for more detailed methods see van der Meer and Redish (2011).

### Identifying consistent cells across days

In order to identify when the same cell was recorded across several days, we combined techniques previously described by Schmitzer-Torbert and Redish (2004) and Tolias et al. (2007). Briefly, we examined the correlation of the average waveforms recorded for each spike train to all the putative cells recorded on the subsequent day of running. Spike trains that were correlated with a subsequently-recorded putative cell at >0.95 were marked as potentially matched across days.

**Figure 3:**
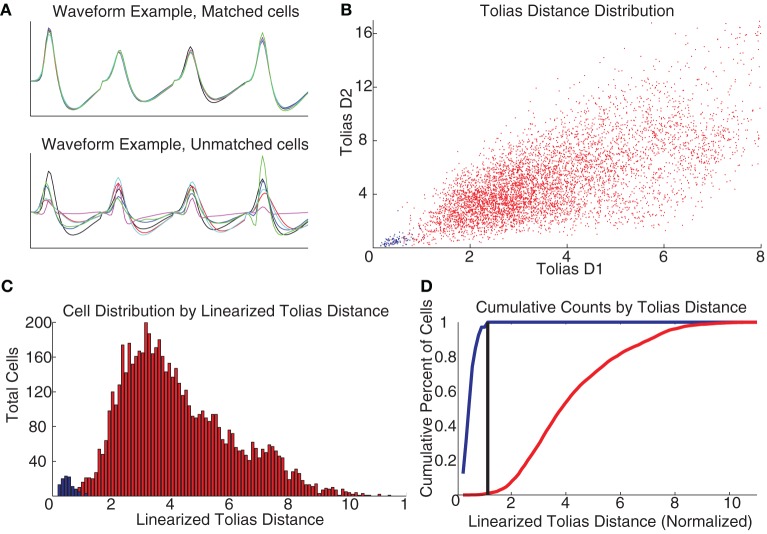
**Recording cells across multiple days**. **(A)** Waveforms were compared across days as per Schmitzer-Torbert and Redish (2004). **(B)** Measuring the *D*1 and *D*2 parameters from Tolias et al. (2007) produces a separation between the putative matched cells (blue) and the unmatched (red), colors the same across all plots. **(C)** Taking the linearized Tolias distance, two distributions are found. **(D)** A classifier was created by placing a decision threshold at the linearized Tolias distance to minimize Type I and Type II errors.

To construct an actual decision-threshold, we calculated the *D*1 and *D*2 measures described by Tolias et al. (2007) for all pairs of spike trains of the 330 total putative cells recorded (independent of whether they were a putative matched pair), and used our set of putative matched cell pairs as a training group to train a classifier similar to the one described by Tolias et al. Figure 3B shows a scatter plot of the two measures for all of our cells, with the putative matched pairs marked in blue and putative unmatched pairs marked in red. Following Tolias et al., this scatter plot was linearized based on the distance between the means of the two distributions. A decision threshold was defined to optimally separate the two groups (see Figure 3). Cells were identified across multiple days based on their relationships across consecutive days, so a cell identified as the same cell from day 1 to day 2 and also from day 2 to day 3 was considered as the same cell across all 3 days.

### Searching for topology of firing correlates in PFC

In order to check for topographical organization of cells by firing correlates in rodent PFC, we used an analysis described by Redish et al. (2001). First, we found firing rates by time for all cells by binning cell firing into 1 sec bins. Then we calculated correlations of these firing rate traces for all pairs of cells recorded on the same tetrode and all pairs of cells recorded on different tetrodes for each day. We then compared these distributions of correlations using a KS-test to see if they significantly differed. We repeated the same procedure for tuning curves calculated over space (32 × 32 bins) and for the 7 × 7 grids of spatial and strategic firing we used for assessing consistency of cell firing across days.

## Results

### Behavior

#### Performance on MT indicates recognition of the change in reward contingency

**Figure 4:**
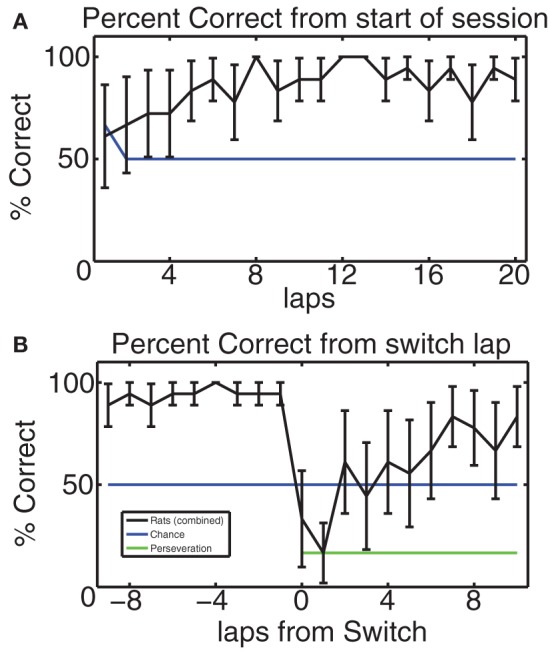
**(A)** Average percent of correct laps over all switch sessions aligned to start of the session. Blue line indicates chance rate of responding. Because the first lap of the alternating contingency was always rewarded, chance is 66% on the first lap, but 50% after that. Rats started at chance and quickly learned to make correct choices. **(B)** Percent of correct laps aligned to the switch. On the switch lap, percent-correct dropped to the expected level given no knowledge of the oncoming switch, but then rose back up to above-chance levels. Blue line indicates chance level of behavior, green line indicates perseveration level.

In order to verify that the rodents did indeed learn the MT-LRA task and respond to its changes in reward contingency, we considered their composite percentage of correct laps for each lap from the start of the session and for 10 laps before and after the switch lap. Figure 4 shows this composite correct percentage for all 6 switch days for all 3 rats. Overall the behavior of our rats was similar to the behavior of rats on this task in previous studies (Schmitzer-Torbert and Redish, 2004; Gupta et al., 2010; van der Meer et al., 2010; Blumenthal et al., 2011; Steiner and Redish, 2012). Animals chose correctly at chance levels for the first several laps (for lap one, chance was 66% because there was a 50% chance of guessing the correct side for either L or R contingencies, but a 100% chance for the A contingency since the first lap was always rewarded for the A contingency). Over the first several laps, rats steadily increased their average percent correct, indicating that they had identified the day's contingency; rats continued to perform above chance until the switch. Figure 4B shows that rats performed above chance on the laps immediately prior to the switch, but that their performance dropped sharply as the reward contingency changed. They collectively performed at perseveration levels for one lap following the switch and then improved steadily to above chance performance, indicating that they correctly identified the new reward contingency. These performance levels are consistent with previous studies of MT-LRA employing the switch sequence (Gupta et al., 2010; Blumenthal et al., 2011; Steiner and Redish, 2012), and indicate that the animals learned to recognize the change in reward contingency.

### Cellular correlates

#### PL firing patterns reflect changes in behavioral strategy

**Figure 5:**
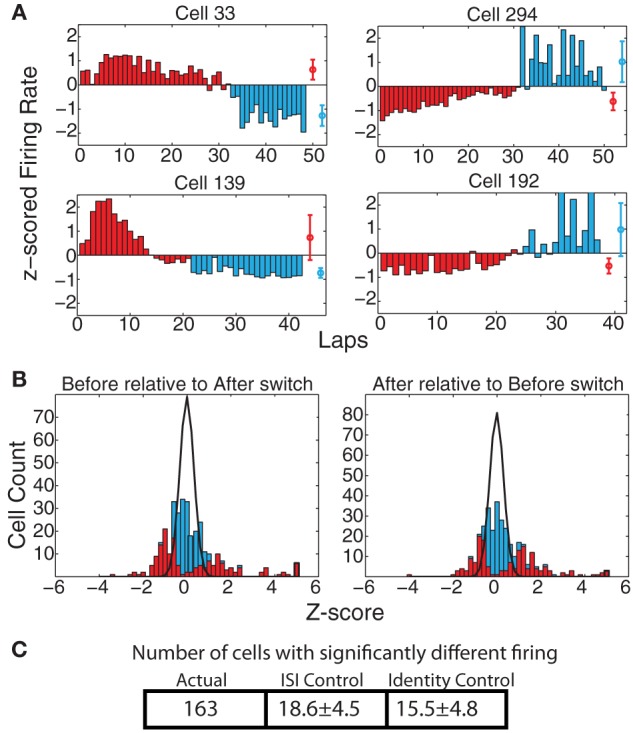
**(A)** Examples of cells with strong changes in firing rate on laps before and after the switch. Red bars indicate laps before the switch, blue bars laps after the switch. Red and blue circles represent average firing rates over both types of laps. **(B)** *z*_after_(before) and *z*_before_(after) comparisons. Bars indicate overall distribution, with red bars indicating cells found to have a significant firing difference by KS test. Black line indicates expected distribution from ID shuffle. **(C)** Number of cells with significantly different firing (by KS test of the distribution of firing rates on laps before the switch compared to after the switch) relative to expected number of cells from two control conditions with inter-spike-intervals (ISI) or lap identity (ID) randomly shuffled.

Prelimbic cortex has been implicated in encoding representations of abstract rules or strategies involved in solving a task (Rich and Shapiro, 2009; Durstewitz et al., 2010). If the firing of the recorded cells encoded strategy in our Multiple-T switch sequence, we would expect to see cells that fire at a different rate on those laps occurring prior to the switch compared to those laps occurring after the switch. Figure 5A shows some examples of such cells. The direction of the change in firing was not consistent (some cells showed higher firing rate before the switch, and others a higher firing rate after the switch), but a significant change was evident in many of the cells recorded. To determine the proportion of cells with significant effects due to strategy shifts, we calculated *z*_after_(before) and *z*_before_(after) (see Methods). These distributions are shown in Figure 5B. Finally, we counted the number of cells with significantly different responses as measured through the KS-test (see Methods). For firing measured over the entire spatial extent of the maze, 163 cells (49.3%) responded significantly differently, while we would have expected only 18.6 ± 4.5 (*SD*) cells under the ISI control and only 15.5 ± 4.8 (*SD*) under the identity control (See Figure 5C). This represents a significantly larger number of cells than would be expected based on either the ISI (*p* = 3 × 10^−225^) or *SD* (*p* = 7 × 10^−212^) shuffles.

The populations of cells with firing rate differences reflecting strategic differences were significantly larger than those expected by chance across all six maze locations: [SoM : (ACTUAL = 75, ISI-control = 10.4 ± 3.3 *p* = 4 × 10^−83^, ID-control = 9.3 ± 3.2 *p* = 10^−96^); NS : (ACTUAL = 88, ISI-control = 12.3 ± 3.4 *p* = 9 × 10^−107^, ID-control = 11.5 ± 3.7 *p* = 2 × 10^−95^); CP : (ACTUAL = 70, ISI-control = 9.3 ± 3.0 *p* = 3 × 10^−93^, ID-control = 8.6 ± 3.0 *p* = 8 × 10^−93^); Top rail : (ACTUAL = 62, ISI-control = 9.7 ± 3.0 *p* = 4 × 10^−67^, ID-control = 8.5 ± 3.0 *p* = 10^−72^); Feeders : (ACTUAL = 148, ISI-control = 18.3 ± 4.4 *p* = 2 × 10^−194^, ID-control = 15.2 ± 4.5 *p* = 10^−190^); bottom rail : (ACTUAL = 75, ISI-control = 14.6 ± 4.0 *p* = 8 × 10^−51^, ID-control = 10.7 ± 3.4 *p* = 4 × 10^−80^)] The difference was strongest at the feeder locations and weakest at the top rail. This discrepancy is not surprising, as the two feeder arms were different in both location and the stimuli animals experienced at each (i.e., flavors, etc). Additionally, animals spent more time in the feeder regions than any other portion of the track, so more firing data was available from this region of the track than any of the others, giving us more power in this region. However, the number of cells differentiating the reward strategy on this task was quite consistent across all maze locations. It is of particular note that the navigation sequence had a similar distribution of strategy-differentiating cells as the rest of the maze. The typical path the animal ran through the navigation sequence was the same before and after the switch even in extreme changes in strategy, such as when most of the laps before the switch were to the left and most of the laps afterwards were to the right. A significant population of cells showing differential firing on the navigation sequence itself implies that these cells are encoding strategic difference and not merely motor-output differences.

In order to ensure that subtle path differences on the navigation sequence were not playing a role in the differential firing reported here, we checked for significant differences in the path the animals took on laps before the switch vs. laps after the switch (see Methods). We found 6 sessions in which there were significant path differences, but excluding these sessions from the analysis made no qualitative difference in the percentage of cells that fired significantly differently to laps before vs. after the switch on either the navigation sequence or any other maze section. Additionally, we checked to see if cells could be responding to differences in running speed in the navigation sequence by regressing the firing rate of all cells in the navigation sequence against the animal's speed through the navigation sequence and removing any cells which had a significant effect. This process left us with a significant population of strategy sensitive neurons: ACTUAL = 55, *p* = 10^−36^ (ISI), *p* = 2 × 10^−32^ (ID).

There is an additional concern that some of the cells we have detected as firing at a different rate before vs. after the switch based on this measure may in fact simply have a continuously variable firing rate that changes over the course of the session. In order to estimate the size of this potential effect, we ran a regression over all cells against lap number and removed any cells which had a significant effect from our population of significantly differentiating cells recorded above. This still left us with a significant population of before vs. after differentiating cells at all maze locations: [Overall: ACTUAL = 109 *p* = 10^−89^ (ISI), *p* = 10^−86^ (ID); SoM: ACTUAL = 65 *p* = 6 × 10^−60^ (ISI), *p* = 5 × 10^−70^ (ID); NS: ACTUAL = 73 *p* = 2 × 10^−69^ (ISI), *p* = 2 × 10^−62^ (*SD*); CP: ACTUAL = 59 *p* = 3 × 10^−63^ (ISI), *p* = 3 × 10^−63^ (ID); Top Rail: ACTUAL = 50 *p* = 10^−40^ (ISI), *p* = 10^−44^ (ID); Feeders: ACTUAL = 106 *p* = 4 × 10^−90^ (ISI), *p* = 3 × 10^−90^ (ID); Bottom Rail: ACTUAL = 63 *p* = 2 × 10^−33^ (ISI), *p* = 10^−53^ (ID)].

**Figure 6:**
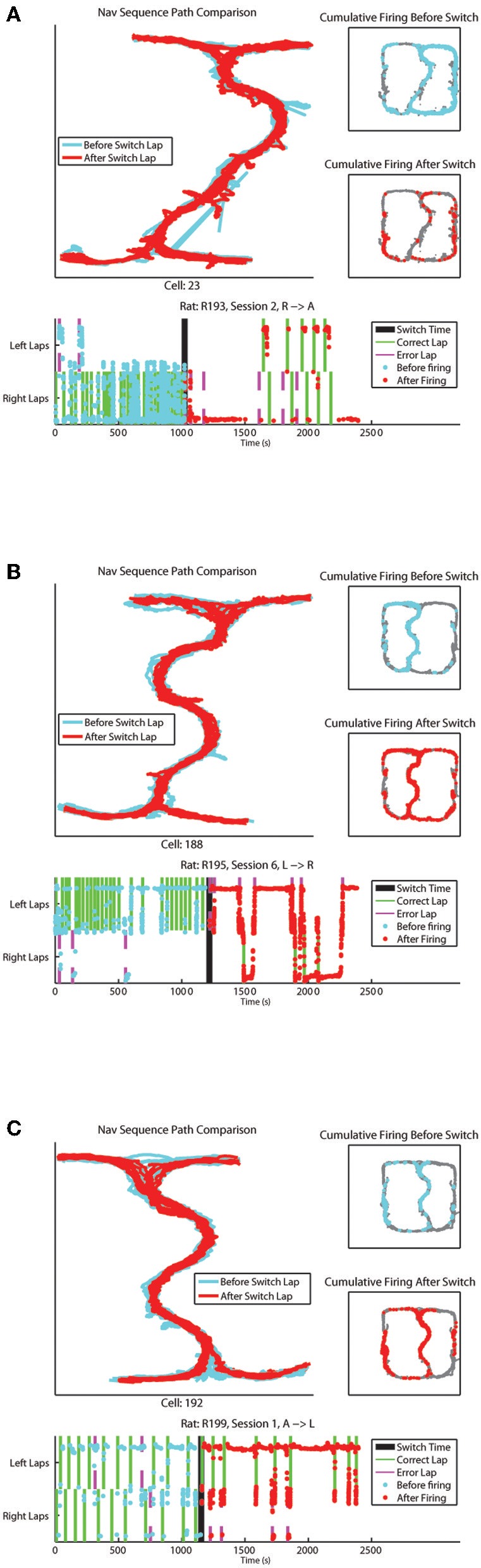
**(A–C)** Represent individual cell firing examples over one session for cells displaying differential before vs. after the switch firing rates. Top left panel compares the animal's path through the Navigation Sequence with laps before the switch in blue and laps after the switch in red. Top right panels display overall spatial firing of the cell before and after the switch respectively. Bottom panel shows the firing of the cell over time of the session, with feeder arrivals for correct and error laps indicated. The y-axis on this plot represents x-position on the track with left and right lap positions marked.

Examples of cells changing their firing patterns in response to strategic considerations before vs. after the switch have been provided in Figure 6. One cell from each rat recorded has been provided, showing combinations of strategies (L, R, and A) before and after the switch.

#### PL firing patterns reflect navigational decisions

**Figure 7:**
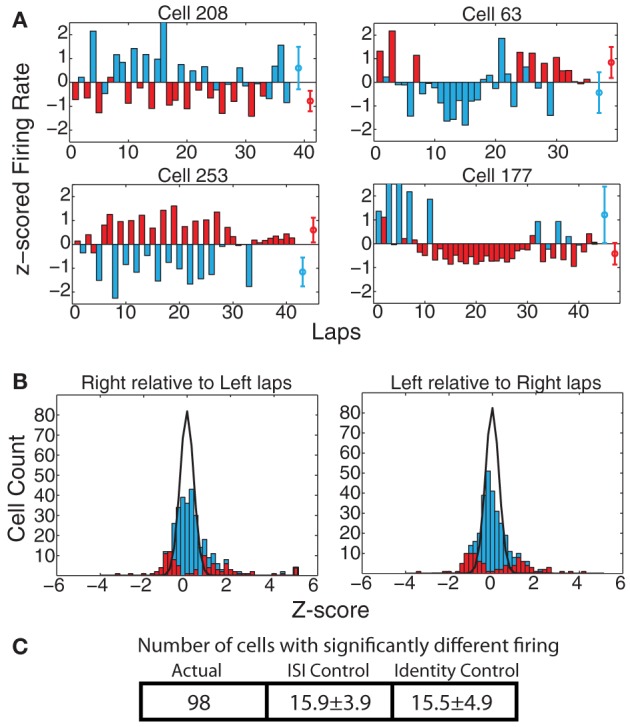
**(A)** Examples of cells with strong changes in firing rate on laps to the left vs. to the right of the track. Red bars indicate laps run to the right, blue bars laps run to the left. Red and blue circles represent average firing rates over both types of laps. **(B)** *z*_right_(left) and *z*_left_(right) comparisons. Bars indicate overall distribution, with red bars indicating cells found to have a significant firing difference by KS test. Black line indicates expected distribution from ID shuffle. **(C)** Number of cells with significantly different firing (by KS test of the distribution of firing rates on laps to the left compared to the right) relative to expected number of cells from two control conditions with inter-spike-intervals (ISI) or lap identity (ID) randomly shuffled.

On the MT-LRA task, each lap has an additional strategic decision question: *Which return-rail is expected to provide food on this lap? Which direction should I go?* As with strategy measures, we found a large proportion of cells that displayed differential firing on left-going and right-going laps. At the population level, 98 cells (29.7%) showed significant differences via the KS-test when measured across the entire spatial extent of the track, which was significantly greater than the expected number of such cells from the ISI-shuffle control (15.9 ± 3.9 (*SD*) *p* = 4 × 10^−100^), and from the identity-shuffle control (15.5 ± 4.9 (*SD*) *p* = 3 × 10^−63^) (see Figure 7).

As with the strategy differences, these navigational differences were significant across all six maze locations. The effect was strongest at the feeders (ACTUAL = 100, ISI-control = 17.0 ± 4.5 *p* = 6 × 10^−76^, ID-control = 15.2 ± 4.7 *p* = 5 × 10^−74^), choice point (ACTUAL = 51, ISI-control = 9.1 ± 3.0 *p* = 10^−45^, ID-control = 8.5 ± 3.1 *p* = 3 × 10^−44^), and top rail (ACTUAL = 81, ISI-control = 10.0 ± 3.2 *p* = 9 × 10^−109^, ID-control = 8.3 ± 3.1 *p* = 10^−125^) and weakest at the start of maze (ACTUAL = 36, ISI-control = 9.7 ± 3.0 *p* = 2 × 10^−18^, ID-control = 9.5 ± 3.3 *p* = 10^−15^) and navigation sequence (ACTUAL = 34, ISI-control = 11.7 ± 3.3 *p* = 6 × 10^−12^, ID-control = 11.4 ± 3.5 *p* = 5 × 10^−11^). However, again, the effect on the start of maze and navigation sequence were both several times more than expected, implying an encoding of navigational plans even before the journeys began to diverge.

As described above, we checked for sessions in which there was a significant difference in the path animals took on laps to the left side of the track vs. laps to the right side. We found 5 such sessions, but excluding these sessions from the analysis did not qualitatively change the percentage of cells that fired significantly differently in response to left-going vs right-going laps on any section of the maze, including the navigation sequence. However, removing speed sensitive cells from the Left/Right sensitive population (as above) dropped that population below significance levels for the Navigation Sequence: ACTUAL = 17, *p* = 0.055 (ISI), *p* = 0.055(ID). This population of neurons is still well above what we typically found from the shuffled data, but could have occurred by chance.

#### PL firing patterns reflect recently-committed errors in behavioral responding

**Figure 8:**
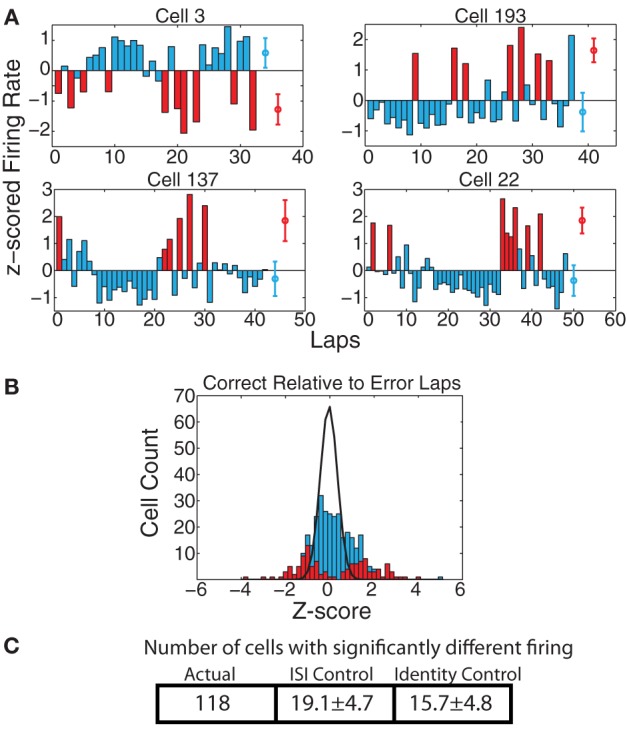
**(A)** Examples of cells with strong changes in firing rate on correct laps vs. laps on which the animal made an error. Red bars indicate error laps, blue bars correct laps. Red and blue circles represent average firing rates over both types of laps. **(B)** *z*_error_(correct) comparison. Because there were too few error laps, we only calculated *z*_error_(correct). Bars indicate overall distribution, with red bars indicating cells found to have a significant firing difference by KS test. Black line indicates expected distribution from ID shuffle. **(C)** Number of cells with significantly different firing (by KS test of the distribution of firing rates on correct laps compared to error laps) relative to expected number of cells from two control conditions with inter-spike-intervals (ISI) or lap identity (ID) randomly shuffled.

Work from Laubach and colleagues (Narayanan and Laubach, 2008; Horst and Laubach, 2009; Narayanan and Laubach, 2009) has shown differential mPFC neural firing on laps following those in which the animal makes a correct decision from those following errors. To check for these effects, we first examined whether there were cells with significant firing rate differences on correct vs. error laps. As shown in Figure 8, a significant portion of cells reflected differences between correct and error laps. We found that, over the full spatial extent of the track, 118 cells (35.8%) had significantly different responding on correct vs. error laps by the KS-test, which was significantly greater than the expected number of such cells from the ISI-shuffle control (19.1 ± 4.7 (*SD*) *p* = 8 × 10^−100^), and from the identity-shuffle control (15.7 ± 4.8 (*SD*) *p* = 3 × 10^−99^).

Unlike the previous two differences examined (before/after switch, left/right), the correct and error laps were only significant for the current lap at the feeder-reward sites (ACTUAL = 131, ISI-control = 24.3 ± 5.5 *p* = 10^−81^, ID-control = 15.6 ± 4.5 *p* = 2 × 10^−142^, not surprising given that correct laps produced food-reward, while error laps did not), and at the bottom section of the maze (ACTUAL = 50, ISI-control = 12.1 ± 3.5 *p* = 5 × 10^−27^, ID-control = 10.6 ± 3.5 *p* = 3 × 10^−29^). As the laps were defined from start of maze to start of maze, the bottom section occurs after food reward, and is, by the Narayanan and Laubach (2008, 2009) and Horst and Laubach (2009) definitions, part of the “next” lap. Thus, we found representations of whether the animal had recently received food (correct vs. error) continuing onto the next lap, consistent with the findings of Narayanan and Laubach (2008, 2009) and Horst and Laubach (2009). In an attempt to determine whether cells with significant firing in this region truly reflect the next lap or just a continuation of the current lap, we further divided the bottom region into two smaller regions, one adjacent to the feeder locations and another adjacent to the Start of Maze. We then checked to see how many of the 50 cells with significant correct vs. error lap firing differences in the bottom region had significant firing differences in either of these sub-regions. We found that 13 cells were significant in the region closer to the feeder and not the other region; 14 cells were significant in the region closer to the Start of Maze and not the other region; 12 cells were significant in both smaller regions; and 11 were not significant in either smaller region by itself. From this we can conclude that firing rate changes due to correct vs. error laps on the bottom rail of the track are no more likely to occur near the feeders than near the Start of Maze region.

We found the largest population of cells with differential firing for correct vs. error laps at the feeder regions, which we expected because of the overt cue of reward receipt (or non–receipt on an error lap) at this region. However, the feeder regions are prone to behavioral confounds because behavior might be quite different at these locations when the animal stops to consume food on correct laps instead of running through with no consumptive behavior on error laps. Due to the setup of our experiment it is difficult to control for all of these factors, but we did check to see if cells had a correlation to running speed by regressing firing rate against running speed for all regions for all cells, and subtracting any cells with significant firing rate modulation to running speed from the counts of cells with significant firing rate modulations for correct vs. error laps. After this subtraction we still had significantly above chance populations on the entire track : ACTUAL = 99 *p* = 4 × 10^−65^ (ISI), *p* = 10^−67^ (ID), the Feeders: ACTUAL = 57 *p* = 2 × 10^−9^ (ISI), *p* = 2 × 10^−20^ (ID), and the Bottom Rail: ACTUAL = 32 *p* = 5 × 10^−9^ (ISI), *p* = 5 × 10^−10^ (ID). While these populations are smaller, they are still significant, even on the bottom rail.

#### These different responding populations are not separate populations

**Figure 9:**
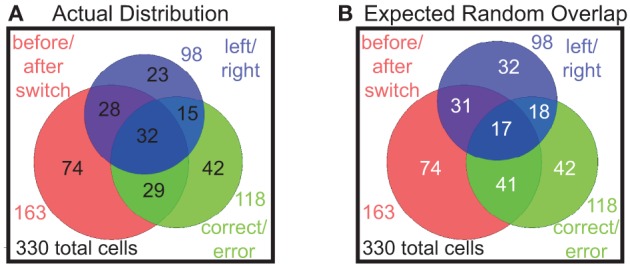
**Distributions of multiple responses**. **(A)** The actual distribution of cells responding to the three key binary factors and the overlap of cells that represent multiple factors. **(B)** Expected distribution overlap if the factors combined independently (i.e., 163/330 = 49% before/after switch responding, 118/330 = 36% correct/error responding, 98/330 = 30% left/right responding).

So far we have found proportions of prelimbic cells that respond to shifts in the reward contingency of laps, errors made in running the task, and to decisions on which direction to go on any given lap. In order to determine whether these were different populations, we examined the overlap of the populations of cells responding significantly to each of these strategies, i.e., the number of cells that responded significantly differently on both correct vs. error laps, as well as laps before vs. after the switch, etc. These results are shown in a Venn diagram in Figure 9. Comparing the actual distribution (Figure 9A) with the expected distribution if the factors combined independently at the same frequency we observed (Figure 9B), we find that these are not distinct populations and that each cell seems to respond to the different binary factors independently, with many cells responding to two or more factors.

#### Spatial firing patterns of PL neurons on the MT-LRA task

**Figure 10:**
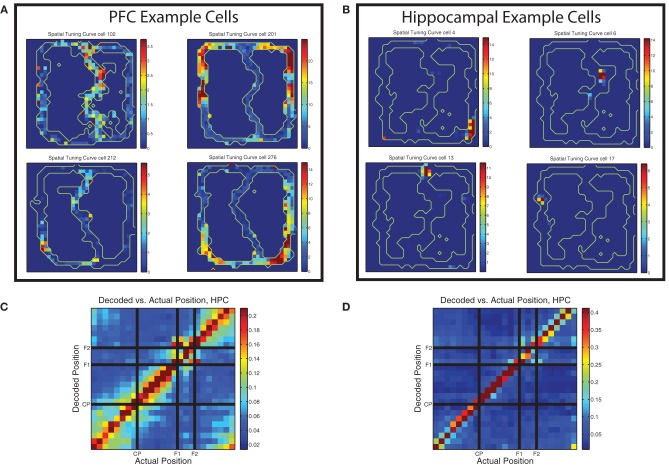
**Prelimbic cells show non–uniform spatial firing**. **(A)** Typical spatial tuning curves of prelimbic cells from this data set. Note the large firing response fields, typically covering a broad region of the maze and usually present on both left and right sides of the track. Color bars indicate firing rate in Hz. **(B)** Typical spatial tuning curves of hippocampal cells from the same task. Note the smaller firing response fields, more typical of hippocampal place cells. Color bars indicate firing rate in Hz. **(C)** Decoding confusion matrix generated from prelimbic cell firing, from 100 random assignments of laps to training and test sets averaged together, then averaged over all rats, all sessions, and left and right laps. Color bar indicates decoded probability at each location. **(D)** Decoding confusion matrix generated from hippocampal cell firing, from 100 random assignments of laps to training and test sets averaged together, then averaged over all rats, all sessions, and left and right laps. Color bar indicates decoded probability at each location.

Early studies of the firing properties of PL neurons in the rat on spatially-based tasks noted the spatial firing properties of these cells (Poucet, 1997; Jung et al., 1998), but most subsequent studies have not focused as directly on the spatial nature of prelimbic cellular correlates (with some exceptions, e.g., Gemmell et al., 2002; Hok et al., 2005; Rich and Shapiro, 2009; de Saint Blanquat et al., 2010). Jung et al. used two restricted spatial tracks (similar to the MT-LRA track) and found that spatial firing patterns in mPFC were markedly different from those of place cells in hippocampus. Spatial tuning curves in PL were often duplicated on topologically similar portions of the track and usually covered larger portions of it than place cells would. In contrast, Poucet used an open field and found no cells that had firing more correlated to space than to specific behaviors. Our task was more similar to the tasks used by Jung et al. and we found similar patterns, with prefrontal cells often firing symmetrically on both sides of the track and usually covering very large portions of it (see Figure 10). This is in contrast to the spatial firing patterns of example hippocampal cells shown in the same figure.

Whether the spatial firing patterns in rodent mPFC actually encode spatial information remains unclear. Several studies in open field environments have failed to find reliable spatial correlates to firing (Poucet, 1997; Gemmell et al., 2002), but others have described place fields in mPFC (Hok et al., 2005). Tasks with more restricted spatial tracks tend to notice non–uniform spatial firing patterns (Jung et al., 1998; Rich and Shapiro, 2009; de Saint Blanquat et al., 2010) similar to what we found. However, in their paper, Jung et al. raised the possibility that spatial firing patterns seen in PL neurons could in fact be a representation of more complex task-based parameters that are correlated with space rather than a representation of space itself, which would coincide with the open field data. The nature of most spatial decision making tasks (such as this one) makes it difficult to conclude whether non–uniform spatial firing patterns of cells are due to a neural representation of the actual location in space, or to a representation of some other task related variable or calculation which happens to coincide with a consistent spatial location. Indeed, because the spatial and task-based strategic components are highly correlated in a task such as ours, it is impossible to completely distinguish these two possibilities in this particular experiment. However, because the cells do exhibit non–uniform firing patterns in space, we ran a decoding analysis to see if we could decode the animal's position from their firing.

There was sufficient spatial information in the prelimbic ensembles to decode position reliably. As shown in Figure 10C, decoding position on the maze from the prelimbic ensembles produced an accurate representation of the current position. Decoding from PL is not as accurate as decoding from HPC (shown in Figure 10D) but in both cases the most likely response is the actual position of the animal. However, the PL decoding shows more off-axis likelihood than the HPC decoding example.

Interestingly, the confusion matrix for prefrontal spatial decoding showed blocks consistent with chunking of the maze, similar to that seen in hippocampal decoding of similar tasks (Gupta et al., 2012). However, as can be seen by comparing Figures 10C,D, there is much heightened off axis spatial decoding in PL, indicating a less accurate spatial representation than HPC, but a much greater degree of spatial chunking. The PL confusion matrix showed heightened off-diagonal decoding for the section of the track prior to the choice point (start of maze to choice point), the section between the choice point and the first feeder, and the section between the second feeder and the start of the maze. The decoding likelihood was low between these sections, but high within each section, indicating that the population of cells in PL treated these different regions of the track as distinct representations. This is consistent with Jung et al.'s observations. Additionally, while the two feeder locations themselves did not confuse with any other region of the track, there was significant similarity in the decoding of each feeder region for the other, indicating that the feeders may be represented similarly to each other, but quite distinctly from the rest of the spatial positions on the track.

The implication of these blocks of consistent firing in PL is that various locations on the task may be represented by different population states in PL. Indeed, the confusion matrix is suggestive of a transition between states, which would imply that different parts of the track have different population representation states in PFC. These states could represent different sub-tasks that are solved at different spatial locations of the track, supporting the view that spatial firing patterns in rodent PL reflect spatially consistent cognitive tasks rather than information about space itself.

While individual cells displayed marked variation in firing over the spatial extent of the track, we did not find evidence that any specific region of the track had an increased firing rate at a population level. To measure this we averaged the firing rate recorded at each maze location (and the firing rate over the entire track) for every cell on every lap we recorded over all rats. These firing rates were markedly similar: (Entire track: 2.7 ± 4.9(*SD*) Hz, Start of Maze: 2.9 ± 6.0(*SD*) Hz, Navigation Sequence: 2.7 ± 5.4(*SD*) Hz, Choice Point: 2.7 ± 5.6(*SD*) Hz, top rail: 2.8 ± 5.9(*SD*) Hz, Feeders: 2.7 ± 5.0(*SD*) Hz, bottom rail: 2.7 ± 5.5(*SD*) Hz). We performed an ANOVA to search for significant group differences in firing rate across these locations and found none (*p* = 0.122).

### Stability of cellular correlates across days

#### Spatial and strategic firing patterns of prelimbic cells are stable across multiple days of running on the track

Given that there were many sub-populations of cells in PFC that responded in different ways, and that these populations appeared to be randomly distributed, an important question is whether a cell's responding patterns remained consistent across sessions on the same task. Building on the methods used previously (Schmitzer-Torbert and Redish, 2004; Tolias et al., 2007), we developed a classifier to assess the consistency of waveforms of pairs of cells recorded on consecutive days so they could be classified into either matched cells (considered to be the same cell recorded across multiple days) or unmatched cells (see Methods and Figure 3 for details). We identified 145 cells recorded individually on just 1 day, and 60 cells recorded across several days. Of the cells recorded across multiple days, 32 were recorded across 2 days, 11 were recorded across 3 days, 5 were recorded across 4 days, 4 were recorded across 5 days, and 8 were recorded across all 6 days of the switch sequence. Having identified cells as being recorded across multiple days, we examined the extent to which their behavioral correlates changed across those multiple days and found them to be remarkably stable.

**Figure 11:**
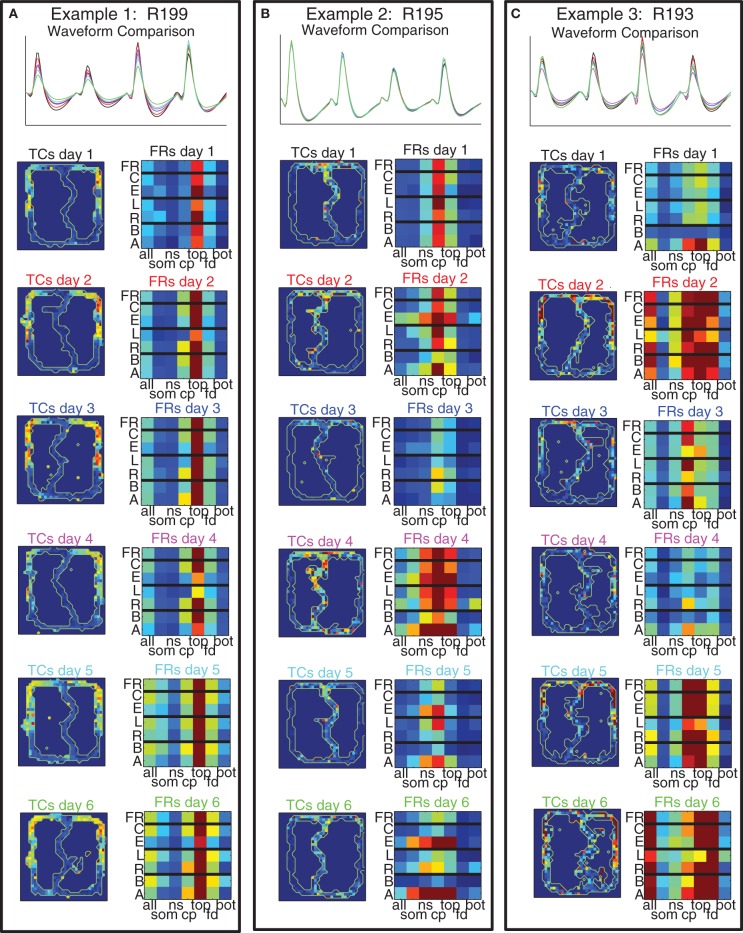
**Example cells with matched waveforms across all 6 days**. For each cell we show: (Top) comparison of waveforms across all 6 days; (Left Column) Spatial tuning curves over the maze for all 6 days; (Right Column) 7 × 7 grid of cell firing. The 7 × 7 grids divide firing rate of the cell into bins dictated by maze location on the x axis (using the 7 maze locations identified in Figure 1) and strategic firing on the y axis [overall firing rate (FR), firing rate on correct laps (C), firing rate on error laps (E), firing rate on laps to the left (L), firing rate on laps to the right (R), firing rate on laps before the switch (B), and firing rate on laps after the switch (A)].

Figure 11 shows three example cells (one from each rat) that we recorded across all 6 days of the switch sequence on the track. The waveforms were well-matched and the spatial tuning curves showed a high degree of similarity across days.

In order to examine the spatial and strategic firing of the cell across an entire session concisely, we created a 7 × 7 grid for each cell for each day with each grid row representing the overall firing rate, firing rate on correct laps and error laps, laps to the left and laps to the right, and laps before and laps after the switch, and each column representing one of the 7 maze locations depicted in Figure 1. (The first column represents firing on the entire lap, columns 2–7 represent the six maze locations.) As with the spatial tuning curves, these plots revealed that strategic firing patterns were highly consistent across days.

**Figure 12:**
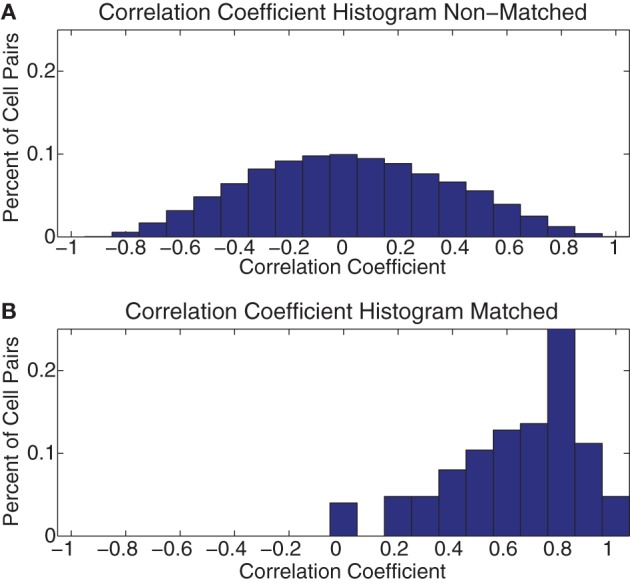
**Histogram of across-day correlations**. **(A)** Histogram of the correlation coefficient of all pairs of the 7 × 7 grids of FR seen in Figure 11 for all pairs of cells not identified as being the same cell recorded across multiple days (Non–Matched). **(B)** The same histogram as in part **(A)** but for all pairs identified as being the same cell on consecutive days (Matched).

In order to determine whether this consistency persisted across all the cells we recorded, we correlated these 7 × 7 grids across days between all pairs of cells. We then separated these correlation coefficients into matched and unmatched cells. As can be seen in Figure 12, the matched cells showed consistent strategic firing patterns, while the unmatched did not. The median of the matched cells was larger than that of the unmatched [Median(matched) = 0.67 ± 0.23 (*SD*), Median(unmatched) = 0.01 ± 0.36 (*SD*)].

From this evidence we conclude that while the cells in PL demonstrated a wide array of firing patterns to many behavioral parameters, the responses of each neuron were highly consistent from day to day.

### Topology of firing correlates in mPFC

#### Prelimbic cells show no evidence for spatial topology of firing correlates

**Figure 13:**
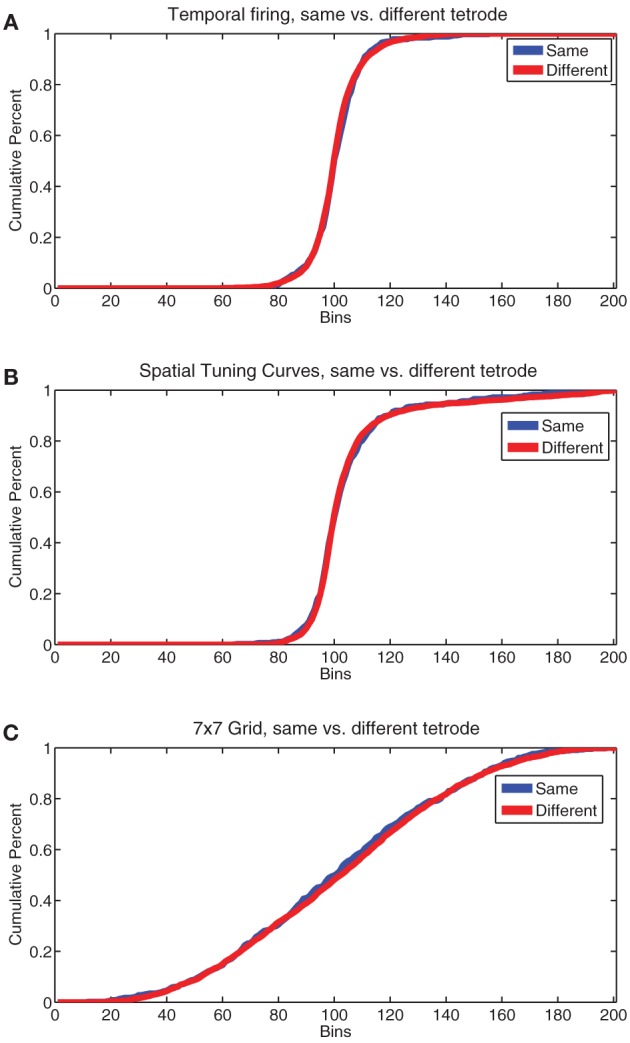
**Comparisons of cell firing patterns on the same vs different tetrodes**. **(A)** Cumulative sum of the normalized histogram of the correlation coefficient of temporal firing rates for all pairs of cells recorded from the same tetrode (same) vs. all pairs of cells recorded from different tetrodes (different). **(B)** Cumulative sum of the normalized histogram of the correlation coefficient of spatial tuning curves for all pairs of cells recorded from the same tetrode (same) vs. all pairs of cells recorded from different tetrodes (different). **(C)** Cumulative sum of the normalized histogram of the correlation coefficient of all pairs of the 7 × 7 grids of FR seen in Figure 11 for all pairs of cells recorded from the same tetrode (same) vs. all pairs of cells recorded from different tetrodes (different).

In humans, research has indicated the presence of topographical patterns of responding in the mPFC from anterior to posterior regions (Koechlin et al., 2003; Koechlin and Summerfield, 2007), not just dorsal to ventral. Recent research into the anterior-posterior distribution of cell populations in rodent PFC has yielded mixed results, but little evidence for an anterior-posterior separation of task-parameter selective cells (Horst and Laubach, 2012, 2013). The fact that many of our single cells responded to several or all of our firing patterns argues against a very distinct topographical grouping. However, to check for topology we used an analysis described in Redish et al. (2001). We compared correlations of pairs of cells recorded on the same tetrode vs different tetrodes on the same day for temporal firing statistics, spatial tuning curves, and the 7 × 7 spatial-strategic grids described above. We found no significant differences in the pattern of correlations for any of these firing patterns (by KS test, temporal firing *p* = 0.18, Tuning Curves *p* = 0.77, 7 × 7 grids *p* = 0.67). Plots of the cumulative sum of histograms of the correlations used for these comparisons are shown in Figure 13. The lack of a significant difference in firing patterns from cells recorded on the same tetrode from those recorded on a different tetrode argues against the presence of a firing-related topology in rodent PL, at least within the spread of our tetrodes (approximately 1 mm centered at 3.0 mm anterior to Bregma).

## Discussion

Prelimbic cells have been reported to have a wide variety of responses to multiple strategic, spatial, and behavioral signals on a variety of tasks. Using a decision-making task that allowed us to study these previously described responses on a single task, we found cells with all of these previously described correlates: Cells in PL had distinct changes in their firing patterns in response to changes in the rule required for solving our task, the future direction the animal was planning to go, and whether the animal had recently made an error or a correct decision. The cells that responded to these different strategic parameters came from different but overlapping populations of cells.

Our results are consistent with most of the previous work on the role of the PL, replicating response-patterns previously described, including strategy-differences (Peyrache et al., 2009; Rich and Shapiro, 2009; Benchenane et al., 2010; Durstewitz et al., 2010), working memory (Yoon et al., 2008; Horst and Laubach, 2009), broad spatial tuning (Jung et al., 1998; Hok et al., 2005; de Saint Blanquat et al., 2010), and post-error differences (Narayanan et al., 2006; Narayanan and Laubach, 2008). Notably, the distinctive spatial firing patterns appeared similar to those previously reported (Jung et al., 1998; Pratt and Mizumori, 2001; Hok et al., 2005; de Saint Blanquat et al., 2010). Although these patterns were very different from the place fields found in hippocampus, they nevertheless provided enough information for accurate spatial decoding; however, it remains likely that the non–uniform firing over space may be caused by firing tuned to cognitive functions that happen to be spatially correlated on this task (Poucet, 1997; Gemmell et al., 2002; Fujisawa et al., 2008). In addition, it is worth noting that the elevated error-related response appeared on the bottom section of the track, which corresponds to the section of the track following encounter with the feeder. This finding is similar to the result, described in Narayanan and Laubach (2008), that cells in PL showed an elevated response following error laps that continued into the start of the next trial.

By matching action potential waveforms across days, we were able to match cells likely recorded across multiple days, and found that both the strategic and spatial firing patterns of the cells were consistent across multiple days on the same task, demonstrating that although there are many potential ways in which cells in PL can respond, the cells do seem to maintain a consistent role on a given task.

Recent work (Rigotti et al., 2013) has discussed the importance of neurons with mixed selectivity encoding in solving cognitive tasks, particularly in the prefrontal cortex of primates. Our current work provides direct evidence for the presence of mixed selectivity neurons in the rodent PL (which is considered to be an analog of prefrontal cortex in primate, Van Eden and Uylings, 1985; Kolb, 1990; Dalley et al., 2004; Kesner and Churchwell, 2011).

PL, along with the infralimbic cortex (IL) and anterior cingulate cortex (ACC), comprise the medial prefrontal cortex of the rat (Van Eden and Uylings, 1985; Kolb, 1990; Dalley et al., 2004; Kesner and Churchwell, 2011). Because of its connectivity, PL is generally thought to be particularly relevant for cognitive functioning (Condé et al., 1995; Vertes, 2004; Goto and Grace, 2005; Jones and Wilson, 2005; Vertes, 2006; Goto and Grace, 2008; Adhikari et al., 2010). Previous work has suggested a potential gradient in the responses of these areas from more motor related in the ACC and dorsal parts of PL (Condé et al., 1995; Vertes, 2004, 2006; Cowen and McNaughton, 2007), to more cognitively related firing in the PL, to more affective and emotional-regulatory actions in the ventral PL and IL regions (Quirk et al., 2000, 2006). Our current recordings were concentrated in PL, where we found multiple, mixed cognitive representations.

## Conclusion

The prefrontal cortex is critical to the ability to integrate multiple dimensions of task-related information in humans (Rushworth et al., 2004), non–human primates (Fuster, 2000), and rats (Dalley et al., 2004; Kesner and Churchwell, 2011). Rigotti et al. (2013) have argued that mixed selectivity-responses in neurons of the non–human primate medial prefrontal cortex are important in efficiently and robustly encoding parameters necessary to solve behavioral tasks. Our findings provide support for this result in the rodent prelimbic cortex. We find that cells in this region responded to many different relevant behavioral parameters of the task, and that many of these cells responded to multiple parameters. Cells also carried spatial information sufficient to decode the animal's position while running on the track, potentially providing for an integration of these task-parameters, goal-information and spatial position. More importantly, we provide the first evidence that the spatial and strategic firing of these cells is consistent across multiple days on the task, indicating that the information being encoded was not simply present for a particular day, but reflected a consistent coding strategy throughout the rats' experience on this particular task.

### Conflict of interest statement

The authors declare that the research was conducted in the absence of any commercial or financial relationships that could be construed as a potential conflict of interest.
